# Undifferentiated Sarcoma: A Rare Tumor of the Prostate

**DOI:** 10.7759/cureus.41056

**Published:** 2023-06-27

**Authors:** Laila Jaouani, Adil Zaimi, Ouissam Al Jarroudi, Sami Aziz Brahmi, Said Afqir

**Affiliations:** 1 Medical Oncology, Mohammed VI University Hospital, Oujda, MAR; 2 Medical Oncology, Faculty of Medicine and Pharmacy, Mohammed First University, Oujda, MAR

**Keywords:** morocco, urinary retention, ki67, immuno-histochemical, anthracycline, undifferentiated sarcoma

## Abstract

Prostate cancer is the most common malignant tumor in men. The vast majority of prostate tumors are represented by prostatic adenocarcinomas (up to 95%). Sarcoma is a very rare tumor in adults with a formidable prognosis. Early diagnosis and radical surgery offer patients the best chance of a cure.

We report the case of a 44-year-old patient with stage VI unresectable high-grade undifferentiated prostate sarcoma, initially presenting with urinary disorders and a large pelvic mass of prostatic origin, with normal Prostate-specific antigen (PSA) levels. The patient was managed by palliative chemotherapy.

## Introduction

Sarcoma represents less than 0.1% of primary prostate cancers [1]. There are several subtypes of this rare histological type, but tumors derived from muscle cells, namely rhabdomyosarcoma in children and leiomyosarcoma in adults, are the most common [2]. The origin of these cancers remains uncertain. They are often discovered at a locally advanced stage due to their non-specific clinical and radiological features; therefore, they generally have a poor prognosis.

## Case presentation

**Figure 1:**
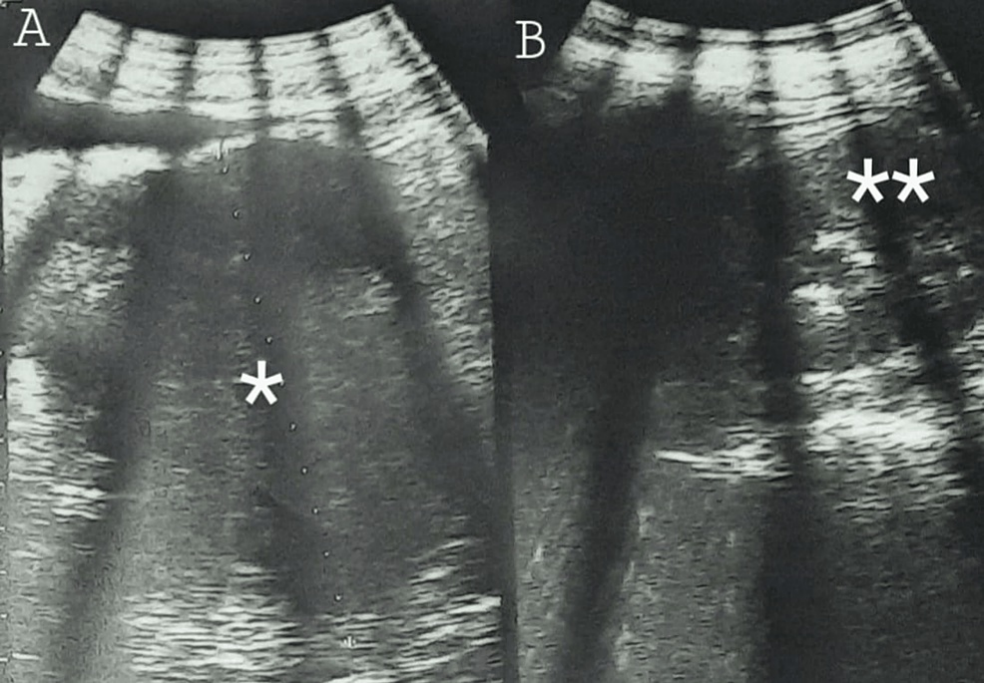
Abdominal-pelvic ultrasound images A: A solid hypoechoic pelvic mass (*). B: A left hydronephrosis (**)

The patient was a 44-year-old man with no prior medical history, who reported urinary disorders (pollakiuria and urge incontinence) and nonspecific abdominal pain for the past four months, evolving in the context of general malaise. Physical examination was unremarkable, save that a digital rectal examination (DRE) revealed an enormous asymmetric prostate gland with a hard mass. During a urology consultation, an abdominal-pelvic ultrasound revealed a huge pelvic mass most likely developed at the expense of the prostate, with left ureterohydronephrosis due to trigonal invasion (Figure 1).

Laboratory tests revealed a Prostate-specific Antigen (PSA) level of 0.93 ng/mL, carcinoembryonic antigen level of 0.5 ng/mL, carbohydrate antigen 19-9 level of 3.8 U/mL, and C-reactive protein level of 7.5 mg/L.

**Figure 2:**
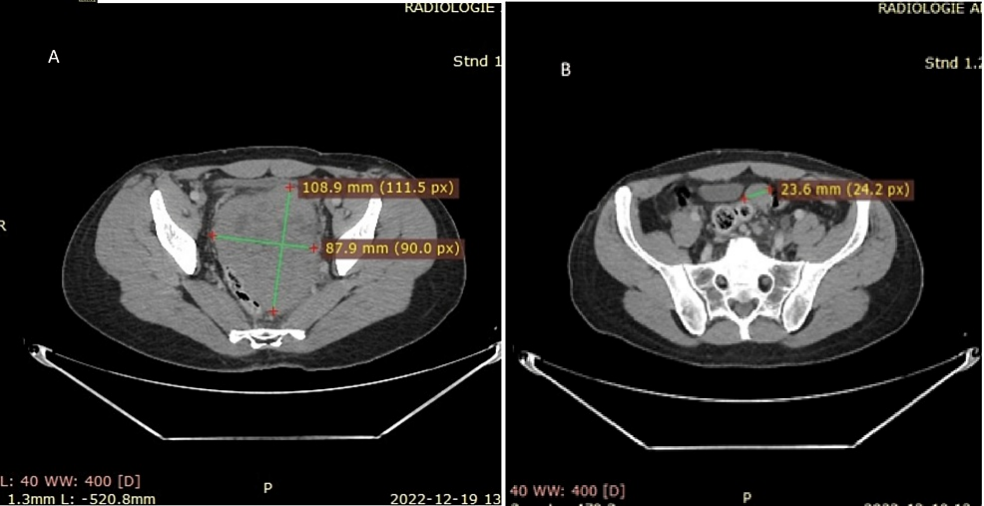
Thoraco-abdominal-pelvic CT scan images A: Large pelvic mass developed at the expense of the prostate; B: Peritoneal nodule

Thoraco-abdominal-pelvic CT scan showed a huge prostatic mass with prostatovesical and prostatorectal shielding and left ureterohydronephrosis. It also showed a nodule in the pelvic peritoneum and ilio-obturator lymphadenopathies (Figure 2).

**Figure 3:**
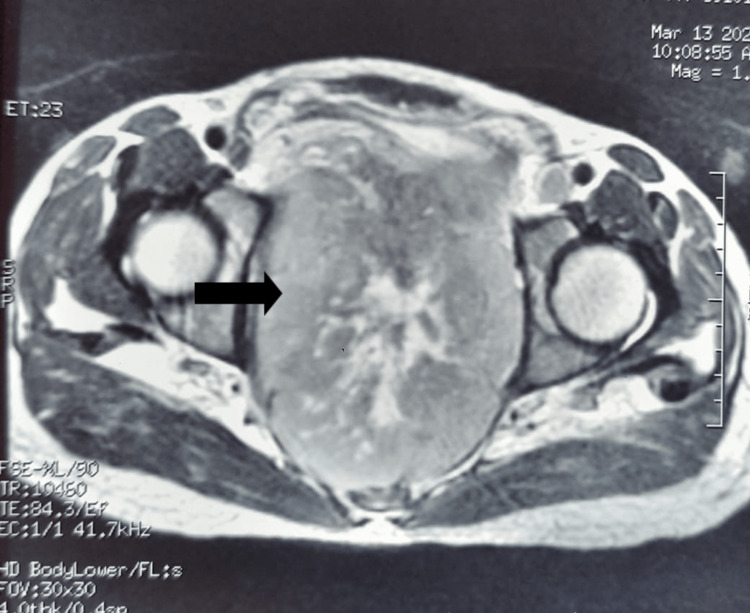
Axial T2-weighted MRI image The image shows a locally advanced solid necrotic pelvic mass (black arrow).

A pelvic magnetic resonance imaging (MRI) was also performed and revealed a large pelvic tumor mass associated with a solid mass in contact with the left colon, causing left ureterohydronephrosis and external iliac vein thrombosis (Figure 3).

**Figure 4:**
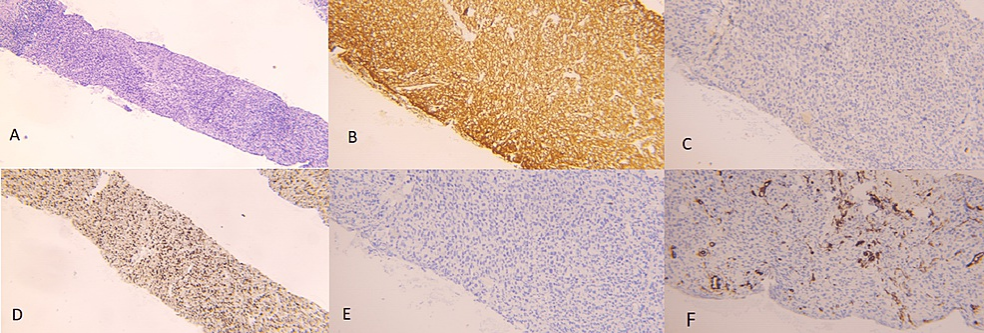
Biopsy of the prostatic mass A: Biopsy displaying a tumoral proliferation made of sheets of cells provided with ovoid, epithelioid, and sometimes fusiform nuclei, with dense chromatin and eosinophilic cytoplasm, arranged within a fibrous stroma with some vascular structures and a discrete lymphocytic contingent. Numerous mitotic figures are present (H&E 4X). B: Tumor cells showing diffuse cytoplasmic expression of SMA (10X). C: Staining by PSA  is negative (10X). D: High proliferative index, ki67 estimated at 80% (H&E 4X). E: Tumoral cells are negative for CKAE1/AE3 (10X). F: CD34 staining of vascular structures, without tumor cell staining (10X). H&E: Hematoxylin and eosin stain.

A transrectal prostate biopsy was performed, and the histopathological examination showed a poorly differentiated malignant tumor proliferation. Immunohistochemistry revealed anti-AML ( actine muscle lisse) antibodies with a proliferation index of 80%, concluding that it was a high grade undifferentiated sarcoma (Table 1, Figure 4).

**Table 1:** Immunohistochemical staining profiles of the prostate tumor cells Anti PSA: Anti-Prostate Specific Antigen; Anti DOG 1: Delay of Germination 1; Anti PS 100: Protein S-100; Anti AML; Anti AML: Actine muscle lisse

Antibodies	Statut
Antibody AE1/AE3	Negative
Antibody anti-PSA	Negative
Antibody anti-Desmin	Negative
Antibody anti-PS100	Negative
Antibody anti-Myogenin	Negative
Antibody anti-CD34	Negative
Antibody anti-DOG1	Negative
Antibody anti-AML	positive
Antibody anti-CD117	Negative
Receptors to estrogen	Negative
Antibody anti-CD45	Negative
Antibody anti-CD20	Negative
Antibody anti-CD3	Negative
Proliferation Index	80% (Ki67)

The case was discussed in a multidisciplinary meeting, and the decision was made to put the patient on palliative chemotherapy, a monotherapy based on fractionated doxorubicin 60 mg/m^2 ^over three days every three weeks was made. A few days later, the patient died of post-chemotherapy tumor lysis syndrome.

## Discussion

Prostate cancer is the most common cancer in men over the age of 50 and is the leading cause of cancer mortality in men over 70 years old [3]. However, prostate sarcomas are rare tumors that can originate from various cellular components and occur at any age, including young individuals [4]. These are malignant tumors developed from mesenchymal tissues (smooth and striated muscle, blood vessels, fibroblasts). These tumors are usually rhabdomyosarcomas, leiomyosarcomas, and specialized prostatic stromal sarcomas. Rhabdomyosarcoma is diagnosed in children between three months and 18 years old. Leiomyosarcoma is the most common in adults with a peak incidence between 40 and 60 years old [5]. Several terms have been used to describe this tumor, including cystosarcoma phyllodes, phyllodes tumor, and atypical stromal hyperplasia [4]. Although radiotherapy is a proven cause of some sarcomas in the irradiated tissue, the origin of these tumors remains uncertain [6].

Clinically, revealing symptoms are irritative lower urinary tract signs, as in our patient, hematuria, dysuria, or acute urinary retention. In 25% of cases, the diagnosis is made at the metastatic stage [7]. Typically, leiomyosarcoma presents as rapidly progressive dysuria in a young man. In the absence of typical clinical signs, sarcoma can be mistakenly diagnosed as benign prostatic hyperplasia.

Laboratory tests (circulating tumor markers) have no specificity, and prostate-specific antigen is usually normal except in carcinosarcomas. Imaging assesses local, regional, or metastatic extension. It includes transabdominal ultrasound and transrectal ultrasound for biopsies. It shows an increase in prostate volume with irregular contours.

Thoraco-abdominal-pelvic CT scan is the key examination for assessing local and regional extension and distant metastases. It usually shows a large solid mass with well or poorly-defined margins with heterogeneous or delayed enhancement, delimiting necrotic-cystic areas [8]. Pelvic MRI is the best way to assess local extension [7].

The diagnosis of prostatic sarcoma is obtained by histopathological and immunohistochemical studies, usually following a transrectal biopsy or transurethral resection [7]. It confirms the mesenchymal origin and determines the subtype of sarcoma. A panel of nonspecific and specific antibodies is available. Cheville et al. in a study of 23 cases of leiomyosarcoma showed that tumor cells were positive in 100% of cases for vimentin, 63% for actin, 20% for desmin, 27% for cytokeratin, and negative for S-100 in all cases [9]. If the subtype of sarcoma cannot be determined, it is called undifferentiated sarcoma. In our case, the tumor cells are only immunoreactive to anti-AML (actine muscle lisse) antibodies with a high proliferation index. 

The treatment of prostatic sarcoma is not yet standardized and involves a multidisciplinary approach, including surgery, pre or post-operative radiotherapy, and neoadjuvant or adjuvant chemotherapy. The choice of treatment depends on various factors, such as the patient's age, histologic type, grade, tumor stage, and the extent of the disease. Polychemotherapy based on anthracyclines is often prescribed, and some phase II randomized clinical trials have shown the benefits of drugs like pazopanib, regorafenib, nivolumab, and imatinib in terms of progression-free survival [10,11]. In our case, due to the patient's poor general condition and the increased risk of tumor lysis syndrome, a monotherapy based on fractionated doxorubicin 60mg/m^2^ over three days was decided, but it was poorly tolerated.

The prognosis of prostatic sarcoma is poor, with reduced survival regardless of surgery [12]. In a multicenter cohort study conducted in China on 41 cases, the average survival was 18.6 months [10]. It is worth noting that some patients die due to disease progression or intolerance to chemotherapy. This was the case with our patient, who died after his first course of chemotherapy due to tumor lysis syndrome.

## Conclusions

Adult prostatic sarcoma is a rare tumor that forms a heterogeneous group of highly malignant tumors with negative PSA (Prostate-specific antigen) and is often diagnosed late. Morphological examination coupled with immunohistochemical studies is key to its diagnosis, while imaging is essential for assessing its extent and post-therapeutic follow-up. Its treatment is not yet standardized, and its prognosis remains poor.

## References

[1] Vandoros GP, Manolidis T, Karamouzis MV (2008). Leiomyosarcoma of the prostate: case report and review of 54 previously published cases. Sarcoma.

[2] Tazi K, el Fassi J, Karmouni T, Koutani A, Hachimi M, Lakrissa A (2001). Prostatic leiomyosarcoma (report of 2 cases) (Article in French). Prog Urol.

[3] Salmi F, Jouhadi H (2018). Étude du profil épidémiologique du cancer de la prostate dans une population marocaine. Revue d’Épidémiologie et de Santé Publique.

[4] Chang YS, Chuang CK, Ng KF, Liao SK (2005). Prostatic stromal sarcoma in a young adult: a case report. Arch Androl.

[5] Hayes-Jordan A, Doherty DK, West SD (2006). Outcome after surgical resection of recurrent rhabdomyosarcoma. J Pediatr Surg.

[6] Scully JM, Uno JM, McIntyre M, Mosely S (1990). Radiation-induced prostatic sarcoma: a case report. J Urol.

[7] Sexton WJ, Lance RE, Reyes AO, Pisters PW, Tu SM, Pisters LL (2001). Adult prostate sarcoma: the M. D. Anderson Cancer Center Experience. J Urol.

[8] Antoun H, Leguen O, Vieillefond A (1997). Imaging of prostatic leiomyosarcoma and contribution of MRI (Article in French). J Radiol.

[9] Cheville JC, Dundore PA, Nascimento AG, Meneses M, Kleer E, Farrow GM, Bostwick DG (1995). Leiomyosarcoma of the prostate. Report of 23 cases. Cancer.

[10] Ding B, Zhang Y, Hu W (2021). Adult primary prostate sarcoma: a multi-center cohort study and comparison between Chinese and American cases. Asian J Surg.

[11] Janet NL, May AW, Akins RS (2009). Sarcoma of the prostate: a single institutional review. Am J Clin Oncol.

[12] von Mehren M, Randall RL, Benjamin RS (2016). Soft tissue sarcoma, version 2.2016, NCCN Clinical Practice Guidelines in Oncology. J Natl Compr Canc Netw.

